# Mutation analysis of the *ATP7B* gene and genotype–phenotype correlation in Chinese patients with Wilson disease

**DOI:** 10.1186/s12876-021-01911-5

**Published:** 2021-09-01

**Authors:** Mingming Li, Jing Ma, Wenlong Wang, Xu Yang, Kaizhong Luo

**Affiliations:** Department of Infectious Diseases, Institute of Hepatology, The Second Xiangya Hospital, Central South University, Changsha, 410011 Hunan Province China

**Keywords:** Wilson disease, *ATP7B* gene, Mutations, Correlation, Chinese

## Abstract

**Aim:**

To discover the novel *ATP7B* mutations in 103 southern Chinese patients with Wilson disease (WD), and to determine the spectrum and frequency of mutations in the *ATP7B* gene and genotype–phenotype correlation in a large-scale sample of Chinese WD patients.

**Methods:**

One hundred three WD patients from 101 unrelated families in southern China were enrolled in this study. Genomic DNA was extracted from the peripheral blood. Direct sequencing of all 21 exons within *ATP7B* was performed. Subsequently, an extensive study of the overall spectrum and frequency of *ATP7B* mutations and genotype–phenotype correlation was performed in all Chinese patients eligible from the literature, combined with the current southern group.

**Results:**

In 103 patients with WD, we identified 48 different mutations (42 missense mutations, 4 nonsense mutations and 2 frameshifts). Of these, 3 mutations had not been previously reported: c.1510_1511insA, c.2233C>A (p.Leu745Met) and c.3824T>C (p.Leu1275Ser). The c.2333G>T (p.Arg778 Leu) at exon 8, was the most common mutation with an allelic frequency of 18.8%, followed by c.2975C>T (p.Pro992Leu) at exon 13, with an allelic frequency of 13.4%. In the comprehensive study, 233 distinct mutations were identified, including 154 missense mutations, 23 nonsense mutations and 56 frameshifts. Eighty-five variants were identified as novel mutations. The c.2333G>T (p.Arg778 Leu) and c.2975C>T (p.Pro992Leu) were the most common mutations, with allelic frequencies of 28.6% and 13.0%, respectively. Exons 8, 12, 13, 16 and 18 were recognised as hotspot exons. Phenotype–genotype correlation analysis suggested that c.2333G>T (p.Arg778 Leu) was significantly associated with lower levels of serum ceruloplasmin (*P* = 0.034). c.2975C>T (p.Pro992Leu) was correlated with earlier age of disease onset (*P* = 0.002). Additionally, we found that the c.3809A>G (p.Asn1270Ser) mutation significantly indicated younger onset age (*P* = 0.012), and the c.3884C>T (p.Ala1295Val) mutation at exon 18 was significantly associated with hepatic presentation (*P* = 0.048). Moreover, the patients with mixed presentation displayed the initial WD features at an older onset age than the groups with either liver disease or neurological presentation (*P* = 0.039, *P* = 0.015, respectively). No significant difference was observed in the presence of KF rings among the three groups with different clinical manifestations.

**Conclusion:**

In this study, we identified three novel mutations in 103 WD patients from the southern part of China, which could enrich the previously established mutational spectrum of the *ATP7B* gene. Moreover, we tapped into a large-scale study of a Chinese WD cohort to characterise the overall phenotypic and genotypic spectra and assess the association between genotype and phenotype, which enhances the current knowledge about the population genetics of WD in China.

## Introduction

Wilson disease (WD), also known as hepatolenticular degeneration (HLD), is an autosomal recessive inherited disorder of copper metabolism, resulting from pathogenic mutations in the *ATP7B* gene. It is characterised by deficient incorporation of copper into ceruloplasmin and decreased biliary copper excretion, leading to excessive copper accumulation, primarily in the liver, brain and eyes. The toxic deposition of copper in the body results in highly heterogeneous clinical presentations, such as liver impairment, neurological disturbance and/or other derangements [1, 2].

The age of onset ranged from 1 to 72 years. Most of the existing literature regards the worldwide prevalence of WD as approximately 1 in 30,000 to 1 in 50,000, with an estimated carrier rate of 1 in 90 [3]. However, in Korea, where WD is one of the most common inherited metabolic disorders, the carrier frequency and incidence of WD are estimated to be 1 in 88.2 and 1 in 30,778, respectively [4]. In Latvia, the estimated prevalence of WD is 1 in 24,000 cases [5]. Screening of WD in the UK population suggests that the frequency of individuals predicted to carry two mutant pathogenic *ATP* alleles is 1 in 7026, which is considerably higher than the typically reported prevalence [6]. Ceruloplasmin-based screening for WD in the population of Japan suggested a frequency as high as 1 in 1500 [7].

WD is caused by mutations in the *ATP7B* gene, discovered in 1993, that encodes a copper transporting P-type ATPase containing 1465 amino acids [8–10]. It is located on chromosome 13q14.3 and consists of 21 exons and 20 introns. Genetic disorders of the *ATP7B* gene disrupt the synthesis and function of the *ATP7B* protein, and further impair the copper excretion pathway, leading to the abnormal deposition of copper in the body. Currently, there are records of at least 800 distinct disease-causing mutations in the *ATP7B* gene, characterised by a few hotspot mutations and a wide spectrum of rare mutations, with obvious ethnic and regional differences.

Traditionally, the diagnosis of WD mainly depends upon clinical manifestations and conventional biochemical indicators, including elevated 24-h urinary copper, low serum ceruloplasmin and increased hepatic copper content. However, biochemical tests can be misleading, making WD diagnosis difficult [11, 12]. Hence, molecular detection is warranted for establishing a precise and decisive diagnosis of WD, particularly in asymptomatic patients and siblings of the proband in a WD-affected family. Previous studies on mutations in Chinese WD patients have been based on diverse genetic detection methods with different detection rates. Therefore, a consensus has not yet been reached regarding the spectrum and frequency of mutations in the *ATP7B* gene in the Chinese WD population. On the other hand, previous studies have failed to identify WD mutations in a significant number of clinically diagnosed cases, resulting in incomplete understanding of the patterns and frequencies of hotspots in the *ATP7B* gene and controversial correlations between genotypes and phenotypes in the Chinese population with WD.

Here, to obtain the best identification rate and accuracy, we used direct sequencing to detect the WD mutations. This method is considered as the gold standard to identify mutations in molecular genetics [13] and has been documented as having a high detection rate and accuracy [14]. In our study, we first analysed the genotypic profile and determined the novel mutations of 103 cases with WD from the south of China by means of direct sequencing. Subsequently, we conducted a comprehensive literature search for available studies on WD mutations to identify the overall spectrum of *ATP7B* mutations and the mutation hotspots observed in the Chinese WD population, and to explore the potential correlation between genotype and phenotype.

To the best of our knowledge, this is the first study to undertake a comprehensive literature study to identify the molecular genetic features and correlations with clinical phenotypes in a large-scale sample of Chinese WD patients. Understanding the genotypic pattern of WD in China could pave the way for offering diagnostic mutational analysis of WD in the future. Our genetic investigation of WD patients from the southern part of China could extend the previously established spectrum of *ATP7B* mutations, and the comprehensive mutation analysis would enhance the current knowledge about the genotypic and phenotypic profiles of WD in China and provides insights into the association between genotype and phenotype in the Chinese population with WD.

## Material and methods

### Patients

A total of 103 WD patients (66 males and 37 females) from 101 independent families were enrolled in this study, with a mean age at presentation of 18.2 ± 12.9 years. They came from different parts of China (40.9% subjects from Hunan Province, 11.3% from Jiangxi Province, 6.8% from Hubei Province and 4.5% from the northern China). All the patients were identified and diagnosed at the Second Xiangya Hospital in Hunan Province between January 2014 and January 2020. Diagnosis of WD was based on a combination of characteristic clinical symptoms, Kayser–Fleischer (KF) rings, abnormal brain magnetic resonance imaging and biochemical parameters, including low serum ceruloplasmin (< 0.2 g/L), increased urinary copper excretion (> 100 μg/24 h) and high hepatic copper content (> 250 μg/g dry weight). The control group consisted of 37 subjects with neither family history nor clinical features of WD. This study was approved by the local ethics committee, and informed consent was obtained from all recruited subjects or from their parents.

### DNA extraction and amplification

The peripheral venous blood was obtained from the WD patients and controls. Genomic DNA was extracted with a Genomic DNA Purification Kit (Qiagen, Hilden, Germany) according to the manufacturer’s protocol. Genomic DNA was stored at − 20 °C. ALL 21 exons of the WD gene were amplified by polymerase chain reaction (PCR). The amplification was conducted as follows: pre-denaturation at 94 °C for 5 min; 35 cycles of denaturation at 94 °C for 45 s, annealing at 55 °C for 45 s (except exon 2.2 and exon 17), or annealing at 51 °C for 45 s (to exon 2.2), or annealing at 61 °C for 45 s (to exon17) and extension at 72 °C for 1 min, with a final elongation at 72 °C for 10 min.

### DNA sequencing

Direct sequencing of the amplified PCR products was performed to detect the disease-causing mutations and single nucleotide polymorphisms at Sangon Biotech (Shanghai, China), and the sequenced products were compared with published normal sequences deposited in GenBank using a blast search program. Samples showing abnormal results were subjected to bidirectional sequencing.

### Systematic review

A comprehensive literature search of PubMed was carried out for articles published from inception until May 2020. Index terms used were Wilson Disease (Title/Abstract) or hepatolenticular degeneration (Title/Abstract). Papers that described the mutations in the WD gene were handpicked by examining online abstracts. Subsequently, further selection was conducted using the full-length publications. Eligibility of the articles was based on the inclusion criteria: (1) observational studies published as original articles that focused on the genetic analysis of WD patients in China; (2) patient cohorts including both paediatric (< 18 years old) and adult (≥ 18 years old) patients; (3) the 21 exons of the *ATP7B* gene were amplified by PCR; and (4) direct sequencing of the PCR products was performed. We excluded the papers in which the subjects failed to satisfy the foregoing criteria, and which were not written in English. To avoid reporting bias, we manually collected additional relevant studies listed as references of these retrieved articles. This systematic review was conducted in accordance with the PRISMA (preferred reporting items for systematic reviews and meta-analysis) guideline.

A standardised data collection form was utilised to derive the following information: first author, country of origin, year of publication, study design and methods, studied population, gender, age at onset of presentations, WD features (clinical manifestations and biochemical markers), and mutations in *ATP7B* gene. A systematic analysis for the spectrum of the *ATP7B* mutations in China was performed on all Chinese WD patients available from qualifying literature, combined with the current southern group. An analysis for the genotype and phenotype correlation in the large-scale Chinese WD cohorts was also performed.

### Statistical analysis

Allele and genotype frequencies were calculated by the direct count. Statistical analyses were performed using SPSS for Windows (Version 20.0, SPSS, Inc., Chicago, IL, USA). The distributed analysis of numeric variables was carried out at first. Normally distributed variables were expressed as mean and standard deviation and were compared between groups using the Scheffe test. Variables that were not normally distributed have been presented as median and interquartile range (IQR) and were compared between groups using the Mann–Whitney U test. Frequencies of qualitative variables were compared between groups by the chi-squared (X) test. A *P* value less than 0.05 was considered statistically significant. Bonferroni correction was applied in case of pairwise comparisons out of larger groups.

## Results

### Mutation analysis in 103 WD patients

Among the 103 WD patients derived from 101 unrelated families, we have identified 48 distinct mutations, including 42 missense mutations, 4 nonsense mutations and 2 frameshifts, as presented in Table 1. None of these 48 mutations was detected among 68 alleles in healthy individuals. According to the records in the WD mutation database of the University of Alberta (http://www.wilsondisease.med.ualberta.ca/database.asp) and the Human Gene Mutation Database (HGMD) professional (http://www.hgmd.cf.ac.uk), this is the first time that the following mutations have been reported: c.1510_1511insA, c.2233C>A (p.Leu745Met), and c.3824T>C (p.Leu1275Ser). All novel missense variants were tested for the possibility of being pathogenic in nature using PolyPhen-2 software (http://genetics.bwh.harvard.edu/pph2/), with the results indicating that they were significantly more likely to alter protein function, five categories based on ACMG guidelines were used: pathogenic, likely pathogenic, uncertain significance, likely benign, and benign for the variant classification of novel variants using ClinVar database, as shown in Table 1. Moreover, 15 known polymorphisms that do not disrupt *ATP7B* gene function were detected (data is available in Table 2). Mutation analysis of the *ATP7B* gene by direct sequencing of 21 exons yielded a mutation detection rate of 80.7% (163/202). There were eight patients having no detectable mutation, indicating that the remaining mutations were possibly located in the intron or the regulator. In the present study, the c.2333G>T (p.Arg778Leu) at exon 8 was the most frequent mutation, with an allelic frequency of 18.8% (38/202), followed by the c.2975C>T (p.Pro992Leu) at exon 13, with an allelic frequency of 13.4% (27/202).

**Table 1:** Spectrum of mutations in the ATP7B gene of a large-scale sample of Chinese patients with Wilson’s disease

Exon	Nucleotide mutation	Amino acid change	Mutation type	Domain	Allelic frequency in southern cohort	Allelic frequency in large-scale cohort	Pathogenicity	
							PolyPhen-2 Score	Variant classification
**2**	**c.121A>G**	**p.Asn41Asp**	**Missense**	**Before Cu1**	**0**	**0.04% (1/2604)**	**0.029**	**Uncertain significance**
2	c.254G>T	p.Gly85Val	Missense	Cu1	0	0.04% (1/2604)		
**2**	**c.268_271DEL**	**p.Lys90PhefsX10**	**Deletion**	**Cu1**	**0**	**0.04% (1/2604)**	**NA**	**Pathogenic**
2	c.287A>G	p.Asp96Gly	Missense	Cu1	0	0.31% (8/2604)		
2	c.314C>A	p.Ser105X	Nonsense	Cu1	0	0.15% (4/2604)		
**2**	**c.367DELG**	**p.Ala123ProfsX30**	**Deletion**	**Cu1**	**0**	**0.04% (1/2604)**	**NA**	**Pathogenic**
2	c.433G>T	p.Val145Phe	Missense	Cu2	0	0.08% (2/2604)		
**2**	**c.523INSA**	**NA**	**Insertion**	**Cu2**	**0**	**0.08% (2/2604)**	**NA**	**NA**
2	c.525DupA	p.Val176SerfsX28	Insertion	Cu2	0.50% (1/202)	1.11% (29/2604)		
2	c.588C>A	p.Asp196Glu	Missense	Cu2	0.99% (2/202)	0.35% (9/2604)		
**2**	**c.592A>G**	**p.Arg198Gly**	**Missense**	**Cu2**	**0**	**0.04% (1/2604)**	**0.735**	**NA**
2	c.685insA	NA	Insertion	Cu3	0	0.04% (1/2604)		
**2**	**c.695DELC**	**p.Pro232GlnfsX30**	**Deletion**	**Cu3**	**0**	**0.04% (1/2604)**	**NA**	**Likely benign​**
**2**	**c.748G>A**	**p.Gly250Arg**	**Missense**	**Cu3**	**0**	**0.04% (1/2604)**	**0.004**	**NA**
2	c.813DELC	p.Cys271TrpfsX3	Deletion	Cu3	0	0.04% (1/2604)		
**2**	**c.898_902DEL AAGTA**	**NA**	**Deletion**	**Cu3**	**0**	**0.04% (1/2604)**	**NA**	**NA**
2	c.994G>T	p.Glu332X	Nonsense	bet Cu3/Cu4	0	0.42% (11/2604)		
**2**	**c.1057DELC**	**p.Gln353ArgfsX10**	**Deletion**	**bet Cu3/Cu4**	**0**	**0.04% (1/2604)**	**NA**	**NA**
2	c.1162C>T	p.GIn388X	Nonsense	Cu4	0	0.04% (1/2604)		
**2**	**c.1168A>G**	**p.Ile390Val**	**Missense**	**Cu4**	**0**	**0.58% (15/2604)**	**0.019**	**NA**
2	c.EX2 DEL	NA	Deletion	Cu4	0	0.08% (2/2604)		
3	c.1366G>C	p.Val456Leu	Missense	bet Cu4/Cu5	0	0.08% (2/2604)		
**3**	**c.1403_1416DEL**	**p.Ala468GlyfsX33**	**Deletion**	**bet Cu4/Cu5**	**0**	**0.04% (1/2604)**	**NA**	**NA**
**3**	**c.1426G>A**	**p.Ala476Thr**	**Missense**	**bet Cu4/Cu5**	**0**	**0.04% (1/2604)**	**0.002**	**Likely benign**
3	c.1448_1455DEL GAGCAGTG	p.Arg483SerfsX20	Deletion	Cu5	0	0.04% (1/2604)		
**3**	**c.1449_1456DEL**	**p.Arg483SerfsX20**	**Deletion**	**Cu5**	**0**	**0.08% (2/2604)**	**NA**	**NA**
3	c.1470C>A	p.Cys490X	Nonsense	Cu5	0.50% (1/202)	0.38% (10/2604)		
3	c.1492A>T	p.Thr498Ser	Missense	Cu5	0	0.04% (1/2604)		
**3**	**c.1510_1511INSA**	**NA**	**Insertion**	**Cu5**	**0.50% (1/202)**	**0.04% (1/2604)**	**NA**	**NA**
3	c.1516_1517DELAT	NA	Deletion	Cu5	0	0.04% (1/2604)		
3	c.1531C>T	p.Gln511X	Nonsense	Cu5	0	1.38% (36/2604)		
4	c.1544G>T	p.Gly515Val	Missense	Cu5	0	0.04% (1/2604)		
**4**	**c.1545DELT**	**p.Gly515GlyfsX9**	**Deletion**	**Cu5**	**0**	**0.04% (1/2604)**	**NA**	**NA**
**4**	**c.1552_1553DELTC**	**p.Ser518ArgfsX15**	**Deletion**	**Cu5**	**0**	**0.04% (1/2604)**	**NA**	**NA**
4	c.1639C>T	p.Gln547X	Nonsense	Cu5	0	0.04% (1/2604)		
**5**	**c.1745_1746DEL TA**	**NA**	**Deletion**	**Cu6**	**0**	**0.04% (1/2604)**	**NA**	**NA**
**5**	**c.1760C>T**	**p.Thr587Met**	**Missense**	**Cu6**	**0**	**0.04% (1/2604)**	**0.005**	**Likely benign​**
**5**	**c.1782T>A**	**p.Tyr594X**	**Nonsense**	**Cu6**	**0**	**0.04% (1/2604)**	**NA**	**NA**
**5**	**c.1802DELC**	**NA**	**Deletion**	**Cu6**	**0**	**0.04% (1/2604)**	**NA**	**Pathogenic​**
5	c.1803DELC	p.Ser602AlafsX46	Deletion	Cu6	0	0.04% (1/2604)		
5	c.1817T>G	p.Val606Gly	Missense	Cu6	0	0.08% (2/2604)		
5	c.1820DUPA	p.Phe608ValfsX2	Insertion	Cu6	0	0.08% (2/2604)		
5	c.1831G>A	p.Glu611Lys	Missense	Cu6	0	0.04% (1/2604)		
5	c.1846C>T	p.Arg616Trp	Missense	Cu6	0	0.04% (1/2604)		
6	c.1875_1876INS AATT	NA	Insertion	Cu6	0	0.04% (1/2604)		
**6**	**c.1925A>G**	**p.Asp642Gly**	**Missense**	**bet Cu6/TM1**	**0**	**0.04% (1/2604)**	**0.899**	**NA**
7	c.1950G>A	p.Trp650X	Nonsense	bet Cu6/TM1	0	0.04% (1/2604)		
**7**	**c.1994T>G**	**p.Met665Arg**	**Missense**	**TM1**	**0**	**0.04% (1/2604)**	**0.836**	**NA**
**7**	**c.2012_2013INS ATAT**	**NA**	**Insertion**	**TM1**	**0**	**0.04% (1/2604)**	**NA**	**NA**
7	c.2038C>T	p.Gln680X	Nonsense	bet TM1/TM2	0.50% (1/202)	0.12% (3/2604)		
**7**	**c.2043DELC**	**p.Ser681SerfsX15**	**Deletion**	**bet TM1/TM2**	**0**	**0.04% (1/2604)**	**NA**	**NA**
**7**	**c.2075T>C**	**p.Leu692Pro**	**Missense**	**TM2**	**0.50% (1/202)**	**0.08% (2/2604)**	**0.996**	**NA**
7	c.2078C>G	p.Ser693Cys	Missense	TM2	0.50% (1/202)	0.15% (4/2604)		
7	c.2097_2099DELCTT	p.Phe700del	Deletion	TM2	0	0.08% (2/2604)		
7	c.2120A>G	p.Gln707Arg	Missense	TM2	0	0.04% (1/2604)		
8	c.2128G>A	p.Gly710Ser	Missense	TM2	0	0.12% (3/2604)		
8	c.2145C>A	p.Tyr715X	Nonsense	TM2	1.49% (3/202)	0.12% (3/2604)		
**8**	**c.2156A>G**	**p.Tyr719Cys**	**Missense**	**bet TM2/TM3**	**0**	**0.04% (1/2604)**	**0.990**	**NA**
**8**	**c.2157C>A**	**p.Tyr719X**	**Nonsense**	**bet TM2/TM3**	**0**	**0.12% (3/2604)**	**NA**	**Pathogenic**
8	c.2185A>G	p.Met729Val	Missense	bet TM2/TM3	0	0.04% (1/2604)		
8	c.2192T>A	p.Val731Glu	Missense	TM3	0	0.08% (2/2604)		
8	c.2195T>C	p.Leu732Pro	Missense	TM3	0	0.04% (1/2604)		
8	c.2223T>A	p.Tyr741X	Nonsense	TM3	0	0.04% (1/2604)		
**8**	**c.2231C>T**	**p.Ser744Phe**	**Missense**	**TM3**	**0**	**0.04% (1/2604)**	**1.000**	**Uncertain significance​**
**8**	**c.2233C>A**	**p.Leu745Met**	**Missense**	**TM3**	**0.50% (1/202)**	**0.04% (1/2604)**	**0.786**	**NA**
**8**	**c.2251G>T**	**p.Ala751Ser**	**Missense**	**TM3**	**0**	**0.04% (1/2604)**	**0.831**	**Uncertain significance​**
**8**	**c.2261A>G**	**p.Glu754Gly**	**Missense**	**bet TM3/TM4**	**0**	**0.04% (1/2604)**	**0.960**	**Benign​**
8	c.2267C>G	p.Ala756Gly	Missense	bet TM3/TM4	0	0.04% (1/2604)		
8	c.2293G>A	p.Asp765Asn	Missense	TM4	0	0.04% (1/2604)		
8	c.2294A>G	p.Asp765Gly	Missense	TM4	1.49% (3/202)	0.35% (9/2604)		
8	c.2297C>T	p.Thr766Met	Missense	TM4	0	0.08% (2/2604)		
**8**	**c.2298INS C**	**NA**	**Insertion**	**TM4**	**0**	**0.08% (2/2604)**	**NA**	**NA**
**8**	**c.2299INSC**	**p.Pro767ArgfsX28**	**Insertion**	**TM4**	**0**	**0.04% (1/2604)**	**NA**	**NA**
8	c.2302DUPC	NA	Insertion	TM4	0	0.08% (2/2604)		
8	c.2304DUPC	p.Met769HisfsX26	Insertion	TM4	0	1.08% (28/2604)		
8	c.2304DELC	p.Met769CysfsX38	Deletion	TM4	0	0.04% (1/2604)		
8	c.2305A>G	p.Met769Val	Missense	TM4	0	0.12% (3/2604)		
**8**	**c.2308C>T**	**p.Leu770Phe**	**Missense**	**TM4**	**0**	**0.08% (2/2604)**	**1.000**	**NA**
**8**	**c.2316_2317INS CTCTTTGTG**	**p.Val772insLeuPheVal**	**Insertion**	**TM4**	**0**	**0.04% (1/2604)**	**NA**	**Uncertain significance​**
8	c.2332C>T	p.Arg778Trp	Missense	TM4	0.99% (2/202)	0.19% (5/2604)		
8	c.2333G>T	p.Arg778Leu	Missense	TM4	18.81% (38/202)	28.57% (744/2604)		
8	c.2333G>A	p.Arg778Gln	Missense	TM4	0	1.42% (37/2604)		
8	c.2336G>A	p.Trp779X	Nonsense	TM4	0	0.04% (1/2604)		
**8**	**c.2341G>A**	**p.Glu781Lys**	**Missense**	**TM4**	**0**	**0.04% (1/2604)**	**0.998**	**NA**
9	c.2383C>T	p.Leu795Phe	Missense	bet TM4/Td	0.50% (1/202)	0.08% (2/2604)		
**9**	**c.2390C>T**	**p.Ser797Phe**	**Missense**	**bet TM4/Td**	**0**	**0.04% (1/2604)**	**0.999**	**Uncertain significance**
10	c.2455C>T	p.Gln819X	Nonsense	bet TM4/Td	0	0.04% (1/2604)		
10	c.2464DUPA	p.Met822AsnfsX32	Insertion	bet TM4/Td	0	0.19% (5/2604)		
**10**	**c.2506G>A**	**p.Gly836Arg**	**Missense**	**Td**	**0**	**0.04% (1/2604)**	**0.998**	**NA**
10	c.2509G>T	p.Gly837X	Nonsense	Td	0	0.04% (1/2604)		
10	c.2510DELG	p.Gly837GlufsX35	Deletion	Td	0	0.04% (1/2604)		
10	c.2519C>T	p.Pro840Leu	Missense	Td	0	0.04% (1/2604)		
**10**	**c.2525A>G**	**p.Asp842Gly**	**Missense**	**Td**	**0**	**0.04% (1/2604)**	**0.999**	**NA**
10	c.2549C>T	p.Thr850Ile	Missense	Td	1.49% (3/202)	0.23% (7/2604)		
**10**	**c.2561A>G**	**p.Glu854Gly**	**Missense**	**Td**	**0**	**0.04% (1/2604)**	**0.998**	**NA**
10	c.2564C>A	p.Ser855Tyr	Missense	Td	0	0.04% (1/2604)		
**11**	**c.2587C>T**	**p.Pro863Ser**	**Missense**	**Td**	**0**	**0.04% (1/2604)**	**0.950**	**Uncertain significance**
**11**	**c.2593_2594INS GTCA**	**NA**	**Insertion**	**Td**	**0**	**0.04% (1/2604)**	**NA**	**NA**
11	c.2605G>A	p.Gly869Arg	Missense	bet Td/TM5	0	0.15% (4/2604)		
11	c.2620G>C	p.Ala874Pro	Missense	bet Td/TM5	0.50% (1/202)	0.27% (7/2604)		
11	c.2621C>T	p.Ala874Val	Missense	bet Td/TM5	0.50% (1/202)	2.42% (63/2604)		
11	c.2648_2649DEL	p.Val883AlafsX3	Deletion	bet Td/TM5	0	0.04% (1/2604)		
11	c.2659del G	p.Ala887LeufsX14	Deletion	bet Td/TM5	0	0.04% (1/2604)		
11	c.2662A>C	p.Thr888Pro	Missense	bet Td/TM5	1.49% (3/202)	0.61% (16/2604)		
11	c.2668G>A	p.Val890Met	Missense	bet Td/TM5	0	0.12% (3/2604)		
12	c.2740C>T	p.Gln914X	Nonsense	bet Td/TM5	0	0.04% (1/2604)		
12	c.2755C>G	p.Arg919Gly	Missense	bet Td/TM5	2.97% (6/202)	1.76% (46/2604)		
12	c.2755C>T	p.Arg919Trp	Missense	bet Td/TM5	0	0.08% (2/2604)		
12	c.2761A>C	p.Ser921Arg	Missense	bet Td/TM5	0	0.04% (1/2604)		
12	c.2785A>G	p.Ile929Val	Missense	TM5	0	0.04% (1/2604)		
**12**	**c.2790_2792DEL**	**p.Ile930DEL**	**Deletion**	**TM5**	**0**	**0.23% (6/2604)**	**NA**	**Likely pathogenic​**
**12**	**c.2794_2795INSGT**	**p.Ser932CysfsX4**	**Insertion**	**TM5**	**0**	**0.04% (1/2604)**	**NA**	**NA**
12	c.2804C>T	p.Thr935Met	Missense	TM5	2.97% (6/202)	4.45% (116/2604)		
12	c.2810DELT	p.Val937GlyfsX5	Deletion	TM5	0	0.46% (12/2604)		
12	c.2827G>A	p.Gly943Ser	Missense	TM5	00	0.27% (7/2604)		
12	c.2828G>A	p.Gly943Asp	Missense	TM5	0.50% (1/202)	2.04% (53/2604)		
**12**	**c.2848G>T**	**p.Val950Phe**	**Missense**	**bet TM5/TM6**	**0**	**0.04% (1/2604)**	**0.978**	**NA**
**12**	**c.2853_2856DEL**	**p.Gln951HisfsX15**	**Deletion**	**bet TM5/TM6**	**0**	**0.04% (1/2604)**	**NA**	**Pathogenic**
**13**	**c.2885DELC**	**NA**	**Deletion**	**bet TM5/TM6**	**0**	**0.04% (1/2604)**	**NA**	**NA**
13	c.2887C>T	p.Gln963X	Nonsense	bet TM5/TM6	0	0.04% (1/2604)		
13	c.2905C>T	p.Arg969Trp	Missense	TM6	0	0.04% (1/2604)		
13	c.2906G>A	p.Arg969gGln	Missense	TM6	0.99% (2/202)	0.12% (3/2604)		
13	c.2924C>A	p.Ser975Tyr	Missense	TM6	0.50% (1/202)	0.77% (20/2604)		
13	c.2930C>T	p.Thr977Met	Missense	TM6	0	0.08% (2/2604)		
13	c.2939G>A	p.Cys980Tyr	Missense	TM6	0.50% (1/202)	0.12% (3/2604)		
13	c.2944G>A	p.Ala982Thr	Missense	TM6	0	0.04% (1/2604)		
13	c.2957C>T	p.Ser986Phe	Missense	TM6	0	0.08% (2/2604)		
13	c.2975C>T	p.Pro992Leu	Missense	bet TM6/Ph	13.37% (27/202)	13.02% (339/2604)		
13	c.3007G>A	p.Ala1003Thr	Missense	bet TM6/Ph	0.50% (1/202)	0.19% (5/2604)		
13	c.3008C>T	p.Ala1003Val	Missense	bet TM6/Ph	0	0.04% (1/2604)		
**13**	**c.3010C>T**	**p.Gln1004X**	**Nonsense**	**bet TM6/Ph**	**0**	**0.04% (1/2604)**	**NA**	**NA**
**13**	**c.3028A>G**	**p.Lys1010Glu**	**Missense**	**bet TM6/Ph**	**0**	**0.04% (1/2604)**	**0.997**	**Uncertain significance**
13	c.3029INST	p.Lys1010AsnfsX18	Insertion	bet TM6/Ph	0	0.08% (2/2604)		
13	c.3029A>C	p.Lys1010Thr	Missense	bet TM6/Ph	0	0.12% (3/2604)		
13	c.3041C>T	p.Pro1014Leu	Missense	bet TM6/Ph	0	0.04% (1/2604)		
**13**	**c.3044T>C**	**p.Leu1015Pro**	**Missense**	**bet TM6/Ph**	**0**	**0.04% (1/2604)**	**0.999**	**NA**
13	c.3053C>T	p.Ala1018Val	Missense	bet TM6/Ph	0	0.12% (3/2604)		
**13**	**c.3056A>C**	**p.His1019Pro**	**Missense**	**bet TM6/Ph**	**0**	**0.04% (1/2604)**	**0.993**	**NA**
**14**	**c.3083A>G**	**p.Lys1028Arg**	**Missense**	**Ph**	**0**	**0.04% (1/2604)**	**0.914**	**NA**
14	c.3085A>G	p.Thr1029Ala	Missense	Ph	0.50% (1/202)	0.04% (1/2604)		
14	c.3087DELT	p.Gly1030AlafsX91	Deletion	Ph	0	0.04% (1/2604)		
14	c.3089G>A	p.Gly1030Asp	Missense	Ph	0	0.19% (5/2604)		
**14**	**c.3095T>C**	**p.Ile1032Thr**	**Missense**	**Ph**	**0**	**0.04% (1/2604)**	**0.997**	**NA**
**14**	**c.3098C>T**	**p.Thr1033Ile**	**Missense**	**Ph**	**0**	**0.04% (1/2604)**	**0.999**	**NA**
14	c.3104G>T	p.Gly1035Val	Missense	Ph	0	0.04% (1/2604)		
14	c.3121C>T	p.Arg1041Trp	Missense	ATP loop	0	0.08% (2/2604)		
14	c.3122G>C	p.Arg1041Pro	Missense	ATP loop	0	0.08% (2/2604)		
14	c.3140A>T	p.Asp1047Val	Missense	ATP loop	0	0.27% (7/2604)		
14	c.3155C>T	p.Pro1052Leu	Missense	ATP loop	0	0.12% (3/2604)		
14	c.3157DUPC	p.Leu1053ProfsX16	Insertion	ATP loop	0	0.04% (1/2604)		
**14**	**c.3209C>G**	**p.Pro1070Arg**	**Missense**	**ATP loop**	**0.50% (1/202)**	**0.23% (6/2604)**	**1.000**	**NA**
14	c.3221C>T	p.Ala1074Val	Missense	ATP loop	0	0.04% (1/2604)		
14	c.3236G>T	p.Cys1079Phe	Missense	ATP loop	0	0.04% (1/2604)		
**15**	**c.3263T>C**	**p.Leu1088Ser**	**Missense**	**ATP loop**	**0**	**0.15% (4/2604)**	**1.000**	**NA**
**15**	**c.3271T>C**	**p.Cys1091Arg**	**Missense**	**ATP loop**	**0**	**0.04% (1/2604)**	**0.960**	**Uncertain significance**
**15**	**c.3274A>C**	**p.Thr1092Pro**	**Missense**	**ATP loop**	**0**	**0.08% (2/2604)**	**0.832**	**NA**
15	c.3284A>C	p.Gln1095Pro	Missense	ATP loop	0	0.04% (1/2604)		
15	c.3293C>G	p.Pro1098Arg	Missense	ATP loop	0	0.04% (1/2604)		
**15**	**c.3307DELG**	**NA**	**Deletion**	**ATP loop**	**0**	**0.04% (1/2604)**	**NA**	**NA**
15	c.3310T>C	p.Cys1104Arg	Missense	ATP loop	0	0.04% (1/2604)		
15	c.3311G>A	p.Cys1104Tyr	Missense	ATP loop	0	0.04% (1/2604)		
15	c.3316G>A	p.Val1106Ile	Missense	ATP loop	2.97% (6/202)	1.08% (28/2604)		
**15**	**c.3368C>T**	**p.Pro1123Leu**	**Missense**	**ATP loop**	**0**	**0.04% (1/2604)**	**0.001**	**Uncertain significance**
15	c.3376DELC	p.His1126ThrfsX2	Deletion	ATP loop	0	0.04% (1/2604)		
**15**	**c.3377_3378DELAC**	**p.His1126ProfsX3**	**Deletion**	**ATP loop**	**0**	**0.12% (3/2604)**	**NA**	**NA**
16	c.3426G>C	p.Gln1142His	Missense	ATP loop	0.50% (1/202)	1.04% (27/2604)		
16	c.3443T>C	p.Ile1148Thr	Missense	ATP loop	3.47% (7/202)	3.19% (84/2604)		
**16**	**c.3445G>A**	**p.Gly1149Arg**	**Missense**	**ATP loop**	**0**	**0.04% (1/2604)**	**1.000**	**Uncertain significance**
16	c.3446G>C	p.Gly1149Ala	Missense	ATP loop	0.50% (1/202)	0.04% (1/2604)		
16	c.3446G>A	p.Gly1149Glu	Missense	ATP loop	0.50% (1/202)	0.27% (7/2604)		
**16**	**c.3451C>G**	**p.Arg1151Gly**	**Missense**	**ATP loop**	**0**	**0.04% (1/2604)**	**1.000**	**Uncertain significance**
16	c.3451C>T	p.Arg1151Cys	Missense	ATP loop	0	0.08% (2/2604)		
16	c.3452G>A	p.Arg1151His	Missense	ATP loop	0	0.19% (5/2604)		
16	c.3459G>T	p.Trp1153Cys	Missense	ATP loop	0.99% (2/202)	0.19% (5/2604)		
16	c.3502G>C	p.Ala1168Pro	Missense	ATP loop	0	0.04% (1/2604)		
16	c.3517G>A	p.Glu1173Lys	Missense	ATP loop	0.50% (1/202)	0.54% (14/2604)		
16	c.3532A>G	p.Thr1178Ala	Missense	ATP loop	4.95% (10/202)	0.77% (20/2604)		
**17**	**c.3563T>G**	**p.Leu1188Arg**	**Missense**	**ATP loop**	**0**	**0.04% (1/2604)**	**0.998**	**NA**
17	c.3577G>C	p.Ala1193Pro	Missense	ATP loop	0	0.04% (1/2604)		
**17**	**c.3584C>T**	**p.Ala1195Val**	**Missense**	**ATP loop**	**0**	**0.04% (1/2604)**	**0.997**	**Pathogenic**
**17**	**c.3587A>G**	**p.Asp1196Gly**	**Missense**	**ATP loop**	**0**	**0.04% (1/2604)**	**1.000**	**NA**
17	c.3605C>G	p.Ala1202Gly	Missense	ATP loop	0	0.08% (2/2604)		
17	c.3646G>A	p.Val1216Met	Missense	ATP bind	1.49% (3/202)	1.34% (35/2604)		
**17**	**c.3653T>C**	**p.Leu1218Pro**	**Missense**	**ATP bind**	**0**	**0.04% (1/2604)**	**0.999**	**NA**
17	c.3659C>T	p.Thr1220Met	Missense	ATP bind	0	0.04% (1/2604)		
**17**	**c.3677C>T**	**p.Thr1226Ile**	**Missense**	**ATP bind**	**0.50% (1/202)**	**0.08% (2/2604)**	**0.990**	**NA**
**17**	**c.3679G>C**	**p.Ala1227Pro**	**Missense**	**ATP bind**	**0**	**0.04% (1/2604)**	**0.999**	**NA**
17	c.3682A>T	p.Arg1228X	Nonsense	ATP bind	0	0.04% (1/2604)		
**18**	**c.3700DELG**	**p.Val1234LeufsX96**	**Deletion**	**ATP bind**	**0**	**0.23% (6/2604)**	**NA**	**NA**
**18**	**c.3715G>T**	**p.Val1239Phe**	**Missense**	**ATP bind**	**0**	**0.12% (3/2604)**	**0.997**	**NA**
18	c.3716T>G	p.Val1239Gly	Missense	ATP bind	0.50% (1/202)	0.04% (1/2604)		
**18**	**c.3733C>G**	**p.Pro1245Ala**	**Missense**	**ATP hinge**	**0**	**0.04% (1/2604)**	**1.000**	**Uncertain significance**
18	c.3741C>G	p.His1247Gln	Missense	ATP hinge	0	0.04% (1/2604)		
18	c.3744G>C	p.Lys1248Asn	Missense	ATP hinge	0	0.08% (2/2604)		
**18**	**c.3766_3767DUPCA**	**p.Gln1256ProfsX75**	**Insertion**	**ATP hinge**	**0**	**0.04% (1/2604)**	**NA**	**Pathogenic​**
**18**	**c.3767INSCA**	**NA**	**Insertion**	**ATP hinge**	**0**	**0.08% (2/2604)**	**NA**	**Pathogenic​**
**18**	**c.3776G>T**	**p.Gly1259Val**	**Missense**	**ATP hinge**	**0**	**0.12% (3/2604)**	**0.988**	**NA**
**18**	**c.3791T>C**	**p.Met1264Thr**	**Missense**	**ATP hinge**	**0**	**0.04% (1/2604)**	**0.990**	**NA**
**18**	**c.3793G>T**	**p.Val1265Leu**	**Missense**	**ATP hinge**	**0.50% (1/202)**	**0.04% (1/2604)**	**0.980**	**NA**
**18**	**c.3796G>C**	**p.Gly1266Arg**	**Missense**	**ATP hinge**	**0**	**0.04% (1/2604)**	**0.998**	**NA**
18	c.3799G>A	p.Asp1267Asn	Missense	ATP hinge	0	0.04% (1/2604)		
18	c.3802G>A	p.Gly1268Arg	Missense	ATP hinge	0	0.04% (1/2604)		
18	c.3809A>G	p.Asn1270Ser	Missense	ATP hinge	1.98% (4/202)	1.88% (49/2604)		
18	c.3818C>T	p.Pro1273Leu	Missense	ATP hinge	0	0.08% (2/2604)		
18	c.3818C>A	p.Pro1273Gln	Missense	ATP hinge	0	0.15% (4/2604)		
**18**	**c.3824T>C**	**p.Leu1275Ser**	**Missense**	**ATP hinge**	**0.50% (1/202)**	**0.08% (2/2604)**	**1.000**	**NA**
18	c.3836A>G	p.Asp1279Gly	Missense	ATP hinge	0	0.19% (5/2604)		
18	c.3843DUPT	p.Val1282CysfsX22	Insertion	ATP hinge	0	0.08% (2/2604)		
**18**	**c.3848C>T**	**p.Ala1283Val**	**Missense**	**ATP hinge**	**0**	**0.04% (1/2604)**	**1.000**	**Uncertain significance**
**18**	**c.3851_3876DEL**	**NA**	**Deletion**	**ATP hinge**	**0**	**0.08% (2/2604)**	**NA**	**NA**
18	c.3859G>A	p.Gly1287Ser	Missense	ATP hinge	0.99% (2/202)	0.19% (5/2604)		
18	c.3877G>A	p.Glu1293Lys	Missense	ATP hinge	0	0.04% (1/2604)		
18	c.3884C>T	p.Ala1295Val	Missense	bet ATP hinge/TM7	1.98% (4/202)	0.61% (16/2604)		
18	c.3889G>A	p.Val1297Ile	Missense	bet ATP hinge/TM7	0	0.04% (1/2604)		
**18**	**c.3896T>G**	**p.Leu1299Arg**	**Missense**	**bet ATP hinge/TM7**	**0**	**0.04% (1/2604)**	**0.996**	**NA**
**18**	**c.3901_3902INSA**	**p.Arg1301PhefsX3**	**Insertion**	**bet ATP hinge/TM7**	**0**	**0.08% (2/2604)**	**NA**	**NA**
19	c.3955C>T	p.Arg1319X	Nonsense	bet ATP hinge/TM7	0	0.15% (4/2604)		
19	c.3960G>C	p.Arg1320Ser	Missense	bet ATP hinge/TM7	0.50% (1/202)	0.12% (3/2604)		
19	c.3965G>C	p.Arg1322Pro	Missense	bet ATP hinge/TM7	0.50% (1/202)	0.04% (1/2604)		
19	c.3982G>A	p.Ala1328Thr	Missense	TM7	0.99% (2/202)	0.23% (6/2604)		
19	c.4003G>C	p.Gly1335Arg	Missense	TM7	0	0.23% (6/2604)		
**19**	**c.4005_4006INS**	**p.Gly1335INS LXWVA**	**Insertion**	**TM7**	**0**	**0.08% (2/2604)**	**NA**	**NA**
20	c.4043T>A	p.Ile1348Asn	Missense	TM7	0	0.04% (1/2604)		
20	c.4057T>C	p.Trp1353Arg	Missense	TM8	0	0.08% (2/2604)		
20	c.4059G>A	p.Trp1353X	Nonsense	TM8	0	0.04% (1/2604)		
20	c.4064G>A	p.Gly1355Asp	Missense	TM8	0	0.15% (4/2604)		
20	c.4094_4097DELCTGT	p.Ser1365TrpfsX27	Deletion	TM8	0	0.04% (1/2604)		
20	c.4112T>C	p.Leu1371Pro	Missense	TM8	0.50% (1/202)	0.27% (7/2604)		
20	c.4114C>T	p.Gln1372X	Nonsense	TM8	0.50% (1/202)	0.42% (11/2604)		
21	c.4162DELG	p.Ala1388ArgfsX5	Deletion	after TM8	0	0.04% (1/2604)		
21	c.4175T>A	p.Met1392Lys	Missense	after TM8	0	0.04% (1/2604)		
**21**	**c.4272T>G**	**p.Tyr1424X**	**Nonsense**	**after TM8**	**0**	**0.04% (1/2604)**	**NA**	**NA**
21	c.4333G>C	p.Ala1445Pro	Missense	3COOH	0	0.04% (1/2604)		

*TMS* transmembrane domain, *TDS* transduction domain

Novel mutations are highlighted in bold

**Table 2:** Polymorphisms in *ATP7B* identified in 103 WD patients

Exon	Nucleotide change	Polymorphism	Nucleotide sequence	Area of protein	Type	Frequency (%) [Patients (n = 103)]
1	9A>G	Glu3Glu	GAG > GGA	Before Cu1	Missense	89.47
2	870G>C	Val1290Val	GTG > GTC	Cu3	Silent	95.14
2	1216T>G	Ser406Ala	TCT > GCT	Cu4	Missense	87.37
2	1168A>G	Ile390Val	ATA > GTA	Cu4	Missense	0.97
3	1366G>C	Leu456Val	GTG > CTG	Cu4/Cu5	Missense	87.37
8	2310C>G	Leu770Leu	CTC > CTG	TM4	Silent	35.92
10	2495G>A	Arg832Lys	AGG > AAG	TM4/Td	Missense	66.02
12	2855G>A	Arg952Lys	AGA > AAA	TM5	Missense	74.76
12	2785A>G	Ile929Val	ATC > GTC	TM5	Missense	0.97
13	3009G>A	Ala1003Ala	GCG > GCA	Bet TM6/Ph	Silent	3.88
13	2913T>A	Ala971Ala	GCT > GCA	TM6	Silent	0.97
14	3188C>T	Ala1063Val	GCG > GTG	ATP loop	Missense	0.97
16	3419T>C	Val1140Ala	GTC > GCC	ATP loop	Missense	75.73
18	3889G>A	Val1297Ile	GTC > ATC	ATP hinge	Missense	1.94
18	3798G>T	Gly1266Gly	GGG > GGT	ATP hinge	Silent	1.94

**Fig. 1:**
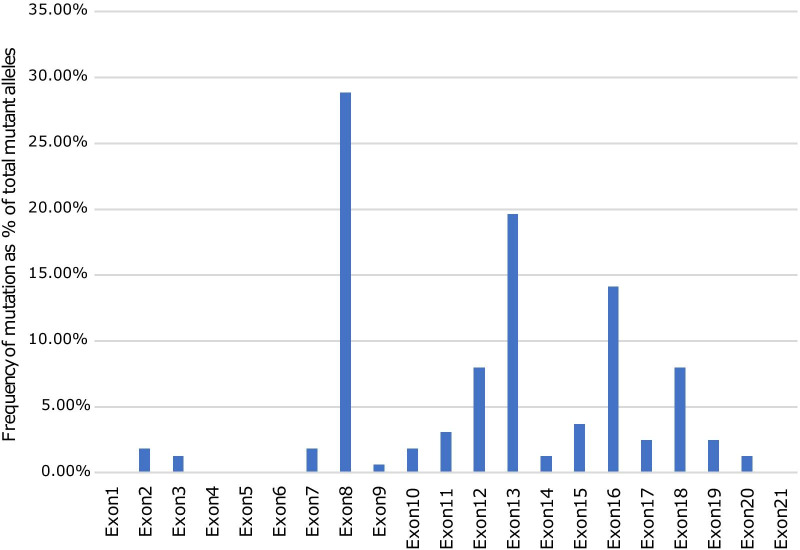
Distribution of mutations in the *ATP7B* gene in patients with Wilson disease (WD). The frequency of mutations found in the cohort of 101 WD index cases is given per exon as a percentage of the total mutant alleles

The exons harbouring the highest percentage of mutations were exons 8, 13, 16, 12 and 18. The total mutation detection rate on these five exons was 63.4% (128/202), suggesting that these exons could be important regions for detecting mutations in the southern Chinese WD cohort. The mutations on exons 8 and 13 accounted for 28.8% (n = 47) and 19.6% (n = 32) of the total mutant alleles (n = 163), respectively. The detection rate of other mutations on exons 16, 12 and 18 spread from 14 to 8.0% (Fig. 1).

### Mutation spectrum of *ATP7B* in a large-scale sample of Chinese patients

**Fig. 2:**
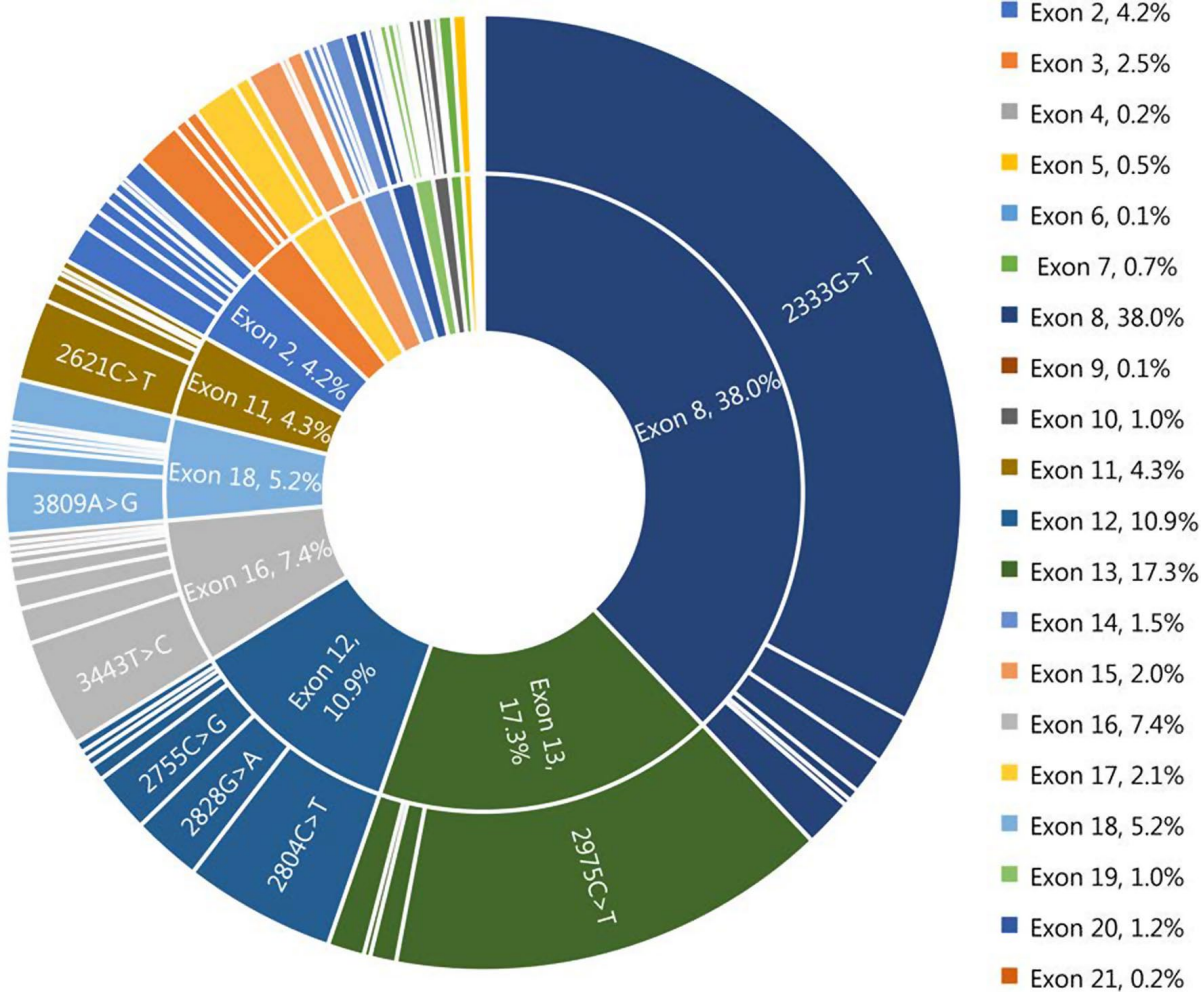
Distribution and frequency of mutations in the *ATP7B* gene in 1302 patients with Wilson disease in the Chinese population

A search of the literature for studies on the overall spectrum of mutations in large-scale sample of Chinese WD patients was conducted. Of the 5,868 studies initially, 5,848 publications were removed due to their irrelevant titles and abstracts. After full-length review of 20 included studies, we further excluded 6 articles that failed to meet the inclusion criteria. Finally, 14 eligible articles with 1201 WD probands were included, and an additional 101 probands from the southern part of China in the present study were also included, for a grand total of 1302 index patients with WD in the systemic analysis. In total, 233 different mutations in the coding region of the ATP7B gene were detected in our patient pool, including 154 missenses, 23 nonsenses and 56 insertions or deletions. Eighty-five variants were identified as novel mutations in the WD databases mentioned above. The computational predictive analysis of the missense variants by PolyPhen-2 was also shown in Table 1. Most of the missense substitutions showed a significant effect on the protein. All mutations accounted for 87.0% (n = 2265) of the alleles studied (n = 2604), with c.2333G>T (p.Arg778Leu) and c.2975C>T (p.Pro992Leu) being the two most common mutations at a frequency of 28.6% and 13.0%, respectively. Mutations were distributed in all exons except exon 1. We found that exons 8, 13, 12 and 16 were the hotspot exons in this large WD pool, accounting for 64.1% (1668/2604) of studied alleles. The mutations accumulated on exons 8, 13, 12 and 16 showed higher prevalence, accounting for 38.0%, 17.3%, 10.9% and 7.4% of the mutant chromosomes, respectively, as depicted in Fig. 2.

It was interesting to note that the prevalent mutation c.2333G>T (p.Arg778Leu) was almost exclusively linked with c.2310C>G (p.Leu770Leu) polymorphism. C.2310C>G (p.Leu770Leu) polymorphism was rare in the normal population but appeared frequently in the WD cohort, suggesting that, to some extent, this linkage may impact the expression of the ATP7B protein.

### Characterisation of clinical phenotype

Screening patients from the 14 eligible reports [14–27] identified 108 patients with clear WD features. We enrolled 196 patients with WD in the final analysis, including the current 88 patients with detailed data from southern China. Of these, 50.5% (99/196) patients had a primary hepatic manifestation, 18.9% (37/196) showed a primary neurological manifestation and 13.8% (27/196) had combined hepatic and neurological manifestations. Thirty-three of the 196 patients (16.8%) presented with no symptoms. The mean age at symptom onset was 19.3 years (from 1 to 62 years). The median ceruloplasmin level was 82.2 mg/L (range 20–962 mg/L).

**Fig. 3:**
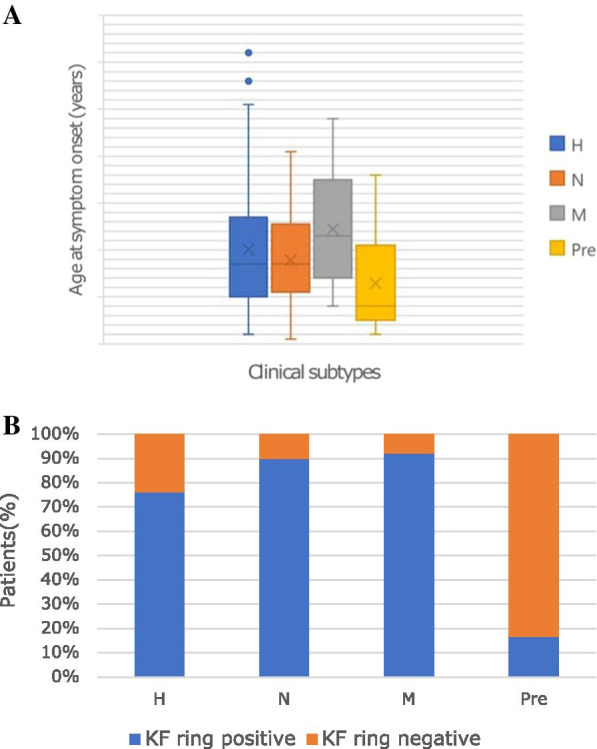
Correlations of four clinical subtypes and **A** onset age and **B** the presence of cornea Kayser–Fleischer (KF) ring. **A** The age of onset was older in the M group than that in the H (*P* = 0.039) and N (*P* = 0.015) groups. The onset age was similar between the H and N groups (*P* > 0.05). The presymptomatic group displayed younger onset age than the H, N and M groups (*P* = 0.000). **B** The presence of KF rings was not significantly different among the three groups with different clinical presentations (*P* > 0.05). *H* hepatic subtype, *N* neurological subtype, *M* mixed subtype, *Pre* presymptomatic subtype

We observed that the patients with mixed manifestation were significantly older at symptom onset than patients with hepatic symptoms (24.4 V 19.4 years of age, *P* = 0.039) and patients with neurological symptoms (24.4 V 17.9 years of age, *P* = 0.015). There is no significant difference in terms of onset age between patients with hepatic phenotype and patients with neurological presentation (*P* > 0.05). In addition, as shown in Fig. 3A, the patients with typically clinical manifestations displayed later age of onset than the clinically asymptomatic patients, which was a statistically significant difference (19.8 V 12.9 years of age, *P* = 0.000). No significant difference was observed in the presence of KF rings among the three clinical subtypes with different manifestations (Fig. 3B).

### Correlation between genotype and phenotype

**Fig. 4:**
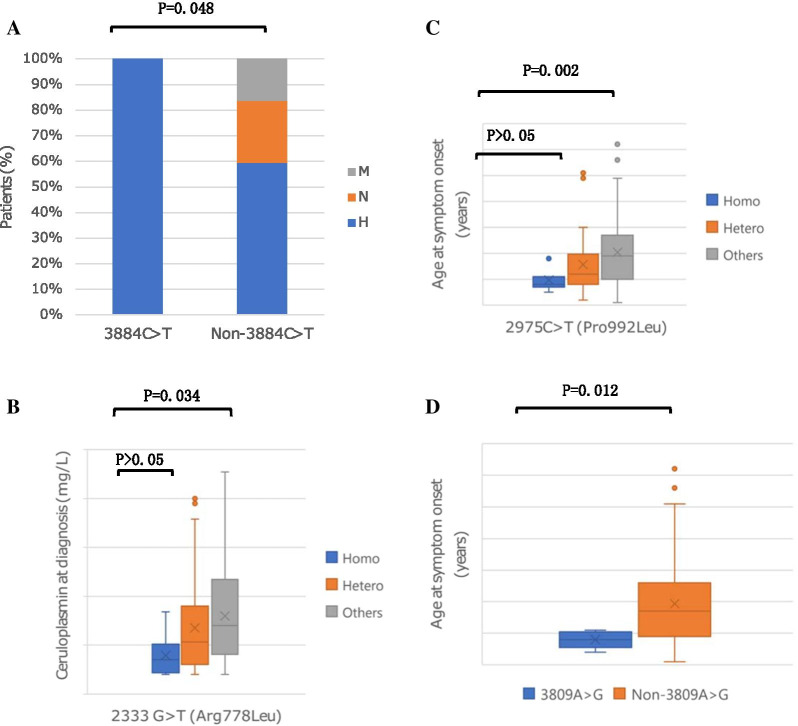
**A** Correlation of 3884C>T (Ala1295Val) and clinical manifestations; **B** correlation of 2333G>T (Arg778 Leu) and serum ceruloplasmin level; **C** correlation of 2975C>T (Pro992Leu) and the onset age; **D** correlation of 3809 A>G (Asn1270Ser) and the onset age. *Homo* homozygotes for the mutation, *Hetero* heterozygotes for the mutation, *H* hepatic manifestation, *N* neurological manifestation, *M* mixed manifestation

To describe the picture of correlation between genotype and phenotype based on the cohort investigation, we initially studied the rarely reported association between the exons and clinical subtypes. First, we examined the hotspot exons in the available 196-patient WD cohort. The results showed that exons 8, 13 and 16 harboured the highest percentage of mutations, consistent with the results described in the large WD patient pool in the comprehensive analysis. Secondly, in different clinical subtypes, we sorted 21 exons in order of mutation frequencies to gain more insights into the most frequent exons in different types of clinical presentations. Notably, in the mixed presentation group, the second most prevalent exon was exon 11, found in 11.5% of mutant alleles, which was much higher than that in the other two groups. Hence, we assumed that the mutations in exon 11 might play an important role in the development of combined presentation. In the primary hepatic group, exon 18 was the third most mutant exon, contributing 10.4% of mutant chromosomes. The possible association between the mutations in exon 18 and the hepatic manifestation is discussed below. In this course, we observed that c.2621C>T (p.Ala874Val), the most prevalent mutation in exon 11, frequently occurred with c.2333 G>T (p.Arg778Leu) except in single heterozygotes. Another observation was that c.3884C>T (p.Ala1295Val), the most prevalent mutation in exon 18, only mutated in patients with hepatic symptoms. The difference between the c.3884C>T (p.Ala1295Val) patients with non-c.3884C>T (p.Ala1295Val) patients in three clinical groups was statistically significant (*P* = 0.048), as shown in Fig. 4A. This indicated that the c.3884C>T (p.Ala1295Val) mutation in exon 18 was significantly associated with hepatic symptoms.

Next, we studied the correlation between specific mutations and the phenotypes. We examined the most prevalent mutations in the available 196-patient WD cohort. The results showed that the two most common mutations were c.2333G>T (p.Arg778Leu) and c.2975C>T (p.Pro992Leu), accounting for 21.4% (84/392) and 12.5% (49/392) of the alleles, in good agreement with the results demonstrated in the comprehensive analysis. With regard to the most frequent mutation, c.2333 G>T (p.Arg778Leu), we observed that patients carrying the c.2333 G>T (p.Arg778Leu) mutation had a lower serum ceruloplasmin levels than patients with other mutations at both alleles. When comparing c.2333 G>T (p.Arg778Leu) homozygous (39.3 ± 23.5 mg/L) or heterozygous patients (67.7 ± 48.1 mg/L) with non-c.2333 G>T (p.Arg778Leu) patients (79.7 ± 47.1 mg/L), we found significant differences (*P* = 0.018, *P* = 0.049, respectively). However, the difference between c.2333G>T (p.Arg778Leu) homozygous and heterozygous patients was not significant, as shown in Fig. 4B. With regard to the second most common mutation, c.2975 C>T (p.Pro992Leu), we found that the patients with c.2975 C>T (p.Pro992Leu) mutation often exhibited symptoms earlier than the patients without c.2975 C>T (p.Pro992Leu)mutation at both chromosomes (Fig. 4C). The difference in the age of onset between c.2975 C>T (p.Pro992Leu) homozygous (9.7 ± 4.2 years of age) and non-c.2975 C>T (p.Pro992Leu) patients (20.5 ± 12.2 years of age) was significant (*P* = 0.01), and the difference between c.2975 C>T (p.Pro992Leu) heterozygous (15.7 ± 11.6 years of age) and non-c.2975 C>T (p.Pro992Leu) patients was also significantly different (*P* = 0.017). No significant difference was observed in the age of onset between c.2975 C>T (p.Pro992Leu) homozygous and heterozygous patients (*P* > 0.05). We also found a dramatic association between the c.3809 A>G (p.Asn1270Ser) mutation and the disease onset age. Statistics showed that the patients with c.3809 A>G (p.Asn1270Ser) mutation had an earlier age of onset (10.8 ± 7.4 years of age) than the non-c.3809 A>G (p.Asn1270Ser) patients (19.3 ± 12.0 years of age) (*P* = 0.012, Fig. 4D).

It should be noted that 82.2% of patients carried at least two mutations at both alleles, while 17.8% patients only carried one mutation at two chromosomes. We did not find a significant correlation between the different forms of mutation (homozygous V heterozygous mutations, combined mutations V single heterozygous mutations) and the several clinical indices in terms of the age of onset, clinical manifestations, ceruloplasmin level and the presence of KF rings.

## Discussion

In the present study, we explored the mutations in the *ATP7B* gene in 101 WD probands from southern China. Forty-eight mutations were found, including 3 novel variants. These novel variants were not found in the control chromosomes. Substituted amino acids with a PolyPhen-2 score close to 1.000 could be predicted to be potentially damaging. However, insertion, deletion and premature stop mutations failed to yield acceptable results from PolyPhen-2 analysis. We regarded the insertion mutation (c.1510–1511 insA) as a clearly pathogenic mutation since it caused a frameshift leading to a premature stop codon. According to the PolyPhen-2 score, the other two variants, c.2233 C>A (p.Leu745Met), c.3824T>C (p.Leu1275Ser)) were both predicted to affect protein function. A mutation detection rate of 80.7% was achieved in the southern cohort, but 19.3% of alleles remained unidentified. One study showed that the rate of mutation detection in this study was 83.8% (67/80) of alleles on direct sequencing of the PCR products of all exons of the ATP7B gene in the 40 unrelated Chinese patients with WD [18]. Rui Hua performed mutational analysis of 68 WD patients from China and found that the rate of mutation detection was up to 97.1% [19]. Failure to detect the remaining mutations may be explained by some objective factors, such as the primers, the PCR procedure and/or the sequence alignment. In our recent studies, the detection rate of direct sequencing could reach as high as 95% with the newly designed PCR primers and the improved amplification requirements. Another reason may be due to the presence of mutations outside the open reading frame of the gene, i.e., in the promoter, introns, the presence of gene rearrangements or possible mutations in other copper-transport chaperone gene. Anna Kluska proved that rare allelic variants in *ESD* and *IN080* increased and decreased the chances for the neurologic phenotype, respectively, while rare variants in *APOE* and *MBD6* decreased the possibilities of WD early manifestation [28]. It was reported that the AmpliSeq Exome kit usually underestimated the insertions and deletions in exome enrichment products [28].

C.2333G>T (p.Arg778Leu) was the most frequent mutation in our study and was also described as the most common mutation in China [16–21, 23, 24], accounting for 18.8% of alleles studied here. The second most common mutation was c.2975 C>T (p.Pro992Leu) among the WD patients, with an allelic frequency of 13.4%, consistent with the frequency previously reported in China [17, 19, 20, 23]. However, Hong et al. [24] suggested that c.3443T>C (p.Ile1148Thr) was the second most common mutation instead of c.2975 C>T (p.Pro992Leu) in their cohort study of 103 Chinese WD patients. An earlier study of 114 WD patients from northern China demonstrated that c.2621 C>T (p.Ala874Val) was the second hot-spot mutation, followed by c.2975 C>T (p.Pro992Leu), at an allelic frequency of 6.1% [29]. We speculate that different gene-level tests or a limited number of patients is largely responsible for the differential conclusions. A large-scale or prospective study, based on the same detection standard, is imperative.

In our current study, exon 8 was the most frequent mutational site, found in 28.8% of mutant alleles, followed by exon 13 in 19.6% and exon 16 in 14.1%, indicating that these three exons could be important regions for detecting mutations.

We conducted a comprehensive analysis of the spectrum and frequency of *ATP7B* mutations in a large-scale sample of Chinese WD patients from more than 30 provinces, autonomous regions and municipalities of China. A total of 233 distinct mutations were detected, of which 85 were novel. The computational predictive analysis software PolyPhen-2 interpreted most of the novel missense variants as disease-causing mutations, with the exception of one benign variant (c.2261A>G, (p.Glu754Gly)). It cannot be ruled out that the silent mutation interpreted as benign could affect protein function.

The most prevalent mutation in the 1302 WD patients pool was c.2333G>T (p.Arg778Leu), in exon 8, with an allelic frequency of 28.6%. The c.2333G>T (p.Arg778Leu) mutation is frequently found in reports of Asian patients, with an allele frequency of 12 to 50% [14, 16–20, 30]. In contrast, the c.3207 C>A (p.His1069Gln) mutation, the most common mutation in European and North American populations, accounting for 30 to 70% of the alleles studied [30], was not detected in any Chinese patients. The next most frequent mutation in this large cohort of Chinese patients was c.2975 C>T (p.Pro992Leu), with an allelic frequency of 13.0%. To our knowledge, the highest frequency of c.2975 C>T (p.Pro992Leu) described so far was 27% [14].

All exons except exon 1 were affected. Notably, exons 8, 13, 12 and 16 were the hot-spot exons identified in the large WD population, accounting for 73.6% of mutant alleles, consistent with previous results that 60.5 to 74% of mutations were located on the above hot-spot exons [31].

The spectrum of WD mutations in the large cohort of Chinese patients consisted of a small number of relatively frequent mutations and a greater number of rare mutations. This further indicated a high degree of mutational heterogeneity, in agreement with previously published findings [32, 33]. Moreover, we found that many mutations were located a short distance away, in line with the preliminary results [34]. Additionally, 64.3% patients were found to stay in a compound heterozygotic state, compared with 13.8% patients in a homozygotic state and 17.4% patients in a single heterozygotic state, which can be explained by the low percentage of consanguinity in our investigated population. No significant difference in phenotypic profiles were found when comparing homozygous or combined heterozygous patients with the patients who had only one mutation at two alleles. We suspected that, to the patients with a single mutation, the remaining unidentified mutations would probably be located in non-coding regions of the *ATP7B* gene. Other mutational mechanisms should also be taken into consideration.

One polymorphism with substitution of leucine with leucine at codon 770 in the transmembrane region of *ATP7B* has been found to be linked with the c.2333 G>T (p.Arg778Leu) mutation. Perhaps, the coexistence of the c.2333G>T (p.Arg778Leu) mutation and the c.2310C>G (p.Arg778Leu) polymorphism would have a special effect on the *ATP7B* protein. Further investigation of the functional implications of both is needed.

A well-defined landscape of the genotype–phenotype correlation will promote the development of clinical studies. However, most of previous studies devoted to genotype–phenotype association have addressed rare or conflicting conclusions [35, 36]. The His1069Gln mutation is most common on Western populations. Genotype–phenotype correlation studies indicated that the His1069Gln mutation was associated more frequently with neurological phenotype [37]. However, the studies in 126 Bulgarian patients presenting a His1069Gln allele frequency in 78% of cases indicated a correlation between that variant and hepatic presentation [38]. Tarnacka et al. reported on 148 Polish patients with a high p.His1069Gln frequency and did not find any association between genotype and phenotype [39]. The studies in Chinese patients showed an association between homozygous p.Arg778Leu and neurologic phenotype [20]. One reported the mutation p.Pro992Leu contributed to early onset age in WD patients, but they did not report any association between p.Arg778Leu mutation and clinical presentation [19]. Verification of this requires a cohort study. Our study significantly described a systemic and quantitative analysis of the genotype–phenotype correlation in a large cohort of Chinese patients with WD.

In the demonstration of hot-spot exons in different types of clinical presentations, we identified that exon 11 was ranked as the second most mutational exon in the mixed presentation. The difference in the proportion of patients with mutations in exon 11 between the hepatic and the mixed group was significant (*P* = 0.046), while the difference between the neurological and the mixed group was not significant. We could not reach the correlation between the mutations in exon 11 and the mixed manifestation. C.2621 C>T (p.Ala874Val), the predominant mutation in exon 11, frequently presented with c.2333 G>T (p.Arg778Leu) substitution. Krishna et al. considered that the hydrophobicity and conformational stability of the hydrophobic domains, such as transmembrane domains, may be altered due to the valine amino acid [40]. We speculated that the transmembrane domain region of ATP7B with valine at the 874 domain region and with leucine at 778 could probably destabilise the formation or influence the expression of protein. Functional studies of mutations are required for the validation of our speculation. Another finding in the analysis of exon hotspots in different clinical presentations was that exon 18 ranked as the third exon with the most mutations in the hepatic presentation group, with a higher mutation frequency (10.38%) than that in the other two groups (6.25% and 3.85% in the neurological and mixed presentation, respectively). This is probably attributable to the potential association between the mutations in exon 18 and the hepatic involvement. Fortunately, we identified that c.3884 C>T (p.Ala1295Val), one kind of the mutations in exon 18, only mutated in the patients with hepatic manifestation. Statistical analysis revealed that there was a significant association between the c.3884 C>T (p.Ala1295Val) mutation and the hepatic phenotype, which was consistent with previous observations that mutations in the conserved ATP hinge region were associated with liver disease without neurological presentation [41], and when the mutation affected the ATP hinge, it resulted in hepatic failure [42].

Furthermore, we found a statistically significant correlation between the c.2333 G>T (p.Arg778Leu) mutation and lower serum ceruloplasmin levels. The difference in the serum ceruloplasmin level between c.2333 G>T (p.Arg778Leu) homozygous or heterozygous patients and non-c.2333 G>T (p.Arg778Leu) patients was significant. A recent study in a large cohort of Chinese WD patients [43] showed that c.2333 G>T (p.Arg778Leu) was related to lower levels of ceruloplasmin as well. That study also suggested that c.2333 G>T (p.Arg778Leu) was related to younger onset age. However, in our study, we did not find the significant difference between c.2333 G>T (p.Arg778Leu) and the onset age, and we did not find a considerable difference between c.2333 G>T (p.Arg778Leu) and the hepatic manifestation either, as previously reported by Liu et al. [44]. Significant difference in the age of onset was observed between c.2975 C>T (p.Pro992Leu) homozygous or combined heterozygous patients and non-2975 C>T (p.Pro992Leu) patients. Collectively, our finding revealed that the patients with c.2975 C>T (p.Pro992Leu) mutation often presented with WD profiles at an earlier age, usually before 13.7 years old, than the patients with other mutations, while Hua et al. [19] described that the patients with c.2975C>T (p.Pro992Leu) often presented WD features before 12 years old. We also found a remarkable association between c.3809 A>G (p.Asn1270Ser) and the disease onset age. Statistical findings showed that the patients with c.3809 A>G (p.Asn1270Ser) mutation usually manifested the WD features before 10.8 years old, much earlier than the patients with other mutations at two chromosomes.

We also observed a visible correlation between the onset age and the characteristic clinical manifestations. Our findings showed that the patients with mixed manifestation had a later age of onset than the groups with either liver disease or neurological phenotype. However, the two latter onset ages were not significantly different from one another, inconsistent with the understanding that patients having predominantly neuropsychiatric symptoms usually manifest symptoms later than patients with hepatic presentation [45–47]. Our results showed that the liver and brain could be affected by WD simultaneously.

In addition, we discovered interesting clinical differences between the symptomatic group and the asymptomatic group in terms of the presence of KF rings. KF rings of symptomatic patients were found to be significantly higher than KF rings of asymptomatic cases, consistent with the earlier findings [48]. We also found that the patients who had KF rings were significantly older at symptom onset than the cohort without KF rings, which is in line with the finding identified in our study that the patients with typical clinical manifestations significantly displayed later age of onset than the patients who were clinically asymptomatic. In summary, the cohort with clinical symptoms presented with a later age of onset and higher prevalence of KF rings than the asymptomatic cohort. One report claimed that patients with purely neurological symptoms were susceptible to KF rings [49]. Our current study did not show any clear difference in the presence of KF rings among the three groups with different clinical manifestations.

In conclusion, we characterised a complete genotypic and phenotypic profile of Chinese patients with WD. The three novel mutations identified in the southern Chinese WD patients could considerably extend the previously established spectrum of the *ATP7B* mutations. Comprehensive mutation analysis will enhance the current knowledge of WD genetics in China. The findings of correlation between specific mutations and clinical features, as well as the age of onset and several clinical profiles provides new insights into the relationships between genotype and phenotype. Additional large studies are required for validation of our conclusions.

## Data Availability

All data generated or analysed during this study are included in this published article, except the sequencing data. All clean sequence data were deposited in the NCBI Sequence Read Archive (SRA, http://www.ncbi.nlm.nih.gov/Traces/sra/) under the accession numbers SRR9969677-SRR9969698.
